# Current and future treatment options for polycythemia vera

**DOI:** 10.1007/s00277-015-2357-4

**Published:** 2015-04-02

**Authors:** Martin Griesshammer, Heinz Gisslinger, Ruben Mesa

**Affiliations:** Johannes Wesling Academic Medical Center, Minden, Germany; Medical University of Vienna, Vienna, Austria; Mayo Clinic Cancer Center, Scottsdale, AZ USA; Schwerpunkte Hämatologie und Onkologie, Hämostaseologie und Palliativmedizin, Johannes Wesling Klinikum Minden, Hans-Nolte-Straße 1, 32429 Minden, Germany

**Keywords:** Polycythemia vera, Hydroxyurea, Interferon, JAK inhibitor, Ruxolitinib

## Abstract

Patients with polycythemia vera (PV), a myeloproliferative neoplasm characterized by an elevated red blood cell mass, are at high risk of vascular and thrombotic complications and have reduced quality of life due to a substantial symptom burden that includes pruritus, fatigue, constitutional symptoms, microvascular disturbances, and bleeding. Conventional therapeutic options aim at reducing vascular and thrombotic risk, with low-dose aspirin and phlebotomy as first-line recommendations for patients at low risk of thrombotic events and cytoreductive therapy (usually hydroxyurea or interferon alpha) recommended for high-risk patients. However, long-term effective and well-tolerated treatments are still lacking. The discovery of mutations in Janus kinase 2 (*JAK2*) as the underlying molecular basis of PV has led to the development of several targeted therapies, including JAK inhibitors, and results from the first phase 3 clinical trial with a JAK inhibitor in PV are now available. Here, we review the current treatment landscape in PV, as well as therapies currently in development.

## Introduction

Polycythemia vera (PV), along with primary myelofibrosis (MF) and essential thrombocythemia (ET), is a classic Philadelphia chromosome-negative myeloproliferative neoplasm (MPN) characterized primarily by an increased red blood cell mass [1–5]. Patients with PV have excessive proliferation of not only erythroid but also myeloid and megakaryocytic components in the bone marrow, resulting in high red blood cell, white blood cell (WBC), and platelet counts [2, 3, 5]. Clinically, patients with PV may experience symptoms such as pruritus, fatigue, night sweats, bone pain, thrombosis, and bleeding [3]. Furthermore, patients with PV have a reduced quality of life and are at risk of transformation to secondary MF and acute myeloid leukemia (AML). Therapeutic options are limited, and available therapies (e.g., low-dose aspirin, phlebotomy, hydroxyurea (HU)) are mainly palliative and focus on preventing the occurrence of thrombosis and improving symptoms.

PV has a much higher prevalence than does MF (44–57 per 100,000 persons vs 4–6 per 100,000, respectively) [6]. In Europe, the incidence of PV ranges from 0.4 per 100,000 persons per year to 2.8 per 100,000 per year [7], and patients with PV have a 1.6-fold higher risk of death than the general population [8]. PV affects more men than women, with the median age of diagnosis being 60 years [8]; however, approximately 20 to 25 % of patients are younger than 40 years [9]. The median survival in patients with PV is 14.1 years, but it is much lower in those older than 60 years and/or with a history of thrombosis (8.3 years) [10].

The understanding of the pathogenesis of PV vastly grew after activating mutations in the Janus kinase 2 (*JAK2*) gene were identified in most patients with PV, with the classical *JAK2* V617F mutation present in approximately 96 % and *JAK2* exon 12 mutations in approximately 3 % of patients with PV [11, 12]. Overactivation of JAK2 autonomously activates downstream pathways, including JAK/STAT, leading to unregulated hematopoiesis. These findings have been instrumental in shaping criteria for diagnosis and treatment, so much that the presence of the *JAK2* V617F mutation is a major criterion in the diagnosis of PV [1] and JAK2 inhibitors are in development as targeted molecular therapies for PV [3, 13].

PV diagnosis is currently based on the 2008 World Health Organization (WHO) diagnostic criteria (Table 1) [1]. The WHO diagnostic criteria emphasize laboratory values, morphologic features, and genetic data, with erythrocytosis being the first major criterion. According to the WHO, evidence of erythrocytosis includes elevated hemoglobin (Hgb) levels (>18.5 g/dL in men and >16.5 g/dL in women), but other groups, such as the British Committee for Standards in Haematology and the Polycythemia Vera Study Group, emphasize the use of elevated hematocrit (Hct) value (>48 % in women and >52 % in men) [14] or red cell mass measurement, respectively [15–17]. Recently, some investigators have proposed revising the WHO criteria [18], especially following the identification of masked PV (mPV) in a subgroup of patients with PV [19]. Unlike patients with overt PV, patients with mPV tend to have normal or borderline Hgb and Hct values but are usually positive for *JAK2* mutations, have bone marrow features consistent with PV, and have low serum erythropoietin levels. Barbui and colleagues [19] stated that a revision to the current WHO diagnostic criteria with emphasis on a lower Hgb threshold and/or the use of Hct threshold values may be helpful in accurately diagnosing those with mPV and could allow for appropriate and prompt treatment of these patients.

**Table 1:** World Health Organization criteria for diagnosing polycythemia vera

Major criteria	Minor criteria
Hgb >18.5 g/dL in men, >16.5 g/dL in women, or other evidence of increased red cell volume^a^	Bone marrow biopsy showing hypercellularity for age with trilineage growth with prominent erythroid, granulocytic, and megakaryocytic proliferation
Presence of *JAK2* V617F or other functionally similar mutations, such as *JAK2* exon 12 mutations	Serum erythropoietin level below the reference range for normal
	Endogenous erythroid colony formation in vitro

Diagnosis requires the presence of both major criteria and one minor criterion or the presence of the first major criterion together with two minor criteria. Republished with permission of the American Society of Hematology, from Vardiman JW et al. [1]; permission conveyed through Copyright Clearance Center, Inc.

*Hct* hematocrit, *Hgb* hemoglobin

^a^Hgb or Hct >99th percentile of method-specific reference range for age, sex, and altitude of residence OR Hgb >17 g/dL in men and >15 g/dL in women if associated with a documented and sustained increase of at least 2 g/dL from a person’s baseline value that cannot be attributed to correction of iron deficiency OR elevated red cell mass >25 % above mean normal predicted value

## Symptom burden and complications of PV

Symptomatic burden in PV is severe and present in most patients with the disease [20]. The most common complaints are fatigue (reported by 88 % of patients), pruritus (62 %), night sweats (52 %), bone pain (50 %), fever (18 %), and weight loss (31 %), with pruritus and fatigue being the most prevalent and troublesome symptoms [3, 20]. Pruritus presents as generalized burning, pricking, tingling, or itching and is frequently reported after water contact (aquagenic pruritus); large temperature shifts, alcohol consumption, or exercise may induce comparable symptoms. Symptoms may persist up to 40 min and are often associated with aggression, irritability, depression, and suicidal ideation. Fatigue has been identified as the consequence of circulating cytokines (tumor necrosis factor alpha, interleukin-1, interleukin-6) [3]. Additionally, approximately 35 to 45 % of patients may develop splenomegaly, although its presence is usually indicative of advanced disease [10]. Splenomegaly usually results in secondary symptoms, including abdominal pain, early satiety, weight loss, and nausea, and complications can lead to abdominal organ compression and portal hypertension [3].

PV-associated constitutional symptoms and symptoms associated with splenomegaly are present in 70 % of patients and compromise quality of life [3, 21], as assessed by tools such as the European Organisation for Research and Treatment of Cancer Quality of Life Questionnaire Core 30 and/or the MPN-Symptom Assessment Form (SAF) questionnaires [20, 21]. An abbreviated version of the MPN-SAF, the MPN-SAF Total Symptom Score, was recently developed to provide an efficient tool for assessing symptom burden in patients with MPN. The MPN-SAF Total Symptom Score is a ten-item scoring instrument focusing on fatigue, concentration, early satiety, inactivity, night sweats, itching, bone pain, abdominal discomfort, weight loss, and fevers [22]. Based on these tools, the symptom burden in patients with PV at diagnosis has been found to be comparable to or worse than that observed in patients with newly diagnosed primary MF [21].

The most frequent complications of PV are vascular and thromboembolic events and hemorrhages [5]. Thrombosis is a prominent symptom observed in up to 39 % of patients with PV at diagnosis [23]. The most frequent types of major thrombosis include stroke, transient ischemic attack, myocardial infarction, peripheral arterial thrombosis, deep venous thrombosis, portal vein thrombosis, and thrombosis of the hepatic veins causing Budd-Chiari syndrome [23, 24]. In addition to macrovascular complications, patients may experience microvascular symptoms (e.g., headaches, dizziness, visual disturbances, distal paresthesia, acrocyanosis), with erythromelalgia being the most characteristic disturbance and consisting of congestion, redness, and burning pain in the extremities [24]. In cases of extreme thrombocytosis (e.g., >1500 × 10^9^/L), patients may be at risk for developing acquired von Willebrand syndrome, which causes a bleeding diathesis [25]. Hemorrhage is also a significant cause of morbidity and mortality in patients with PV [25], with a cumulative incidence of 39.6 % (6.2 % per person-year). Additionally, overall survival has been found to be significantly shorter among patients with hemorrhage than among those without this complication (median overall survival, 94.8 months vs not reached; *P* = 0.002) [25].

PV also carries a risk of transformation into acute leukemia [5]. The incidence of transformation to AML/myelodysplastic syndrome in patients with PV ranges from 5 to 15 % after 10 years of disease, with progressive risk over time [26]. Advanced age; female sex; and the use of alkylating drugs, radiation, or a combination of cytoreductive drugs are associated with a higher risk of leukemic transformation [26].

## Current recommended therapies

Therapeutic options in PV are limited and no cure is available. The goal of current therapies is to prevent the occurrence of thrombosis/vascular events and delay transformation to MF or AML [3, 12, 27, 28]. To this end, treatments for PV aim at targeting an Hct <45 %, as this has been associated with a reduction in cardiovascular deaths and thrombotic events [29–31]. Poorly controlled Hct has been reported to lead to an increased risk of thrombosis because elevated Hct can increase blood viscosity, reduce blood return through the venous system, and increase platelet adhesion [32–34]. A small retrospective landmark study in PV found that the incidence of thrombotic increased linearly in men and women when Hct was >45 % (range, 46–52 %) [35]. More recently, Marchioli et al. tested this recommendation in the Cytoreductive Therapy in Polycythemia Vera (CYTO-PV) study (*N* = 365), a large-scale, prospective, randomized clinical trial comparing the benefits and risks of conventional treatment aimed at maintaining Hct <45 versus 45 to 50 % [36] and found a lower rate of cardiovascular deaths and major thrombotic events in patients with a target Hct of <45 % than in those with a target Hct of 45 to 50 % [31]. The incidence of events was 1.1 per 100 patient-years in the low-Hct group compared with 4.4 per 100 patient-years in the high-Hct group.

**Fig. 1:**
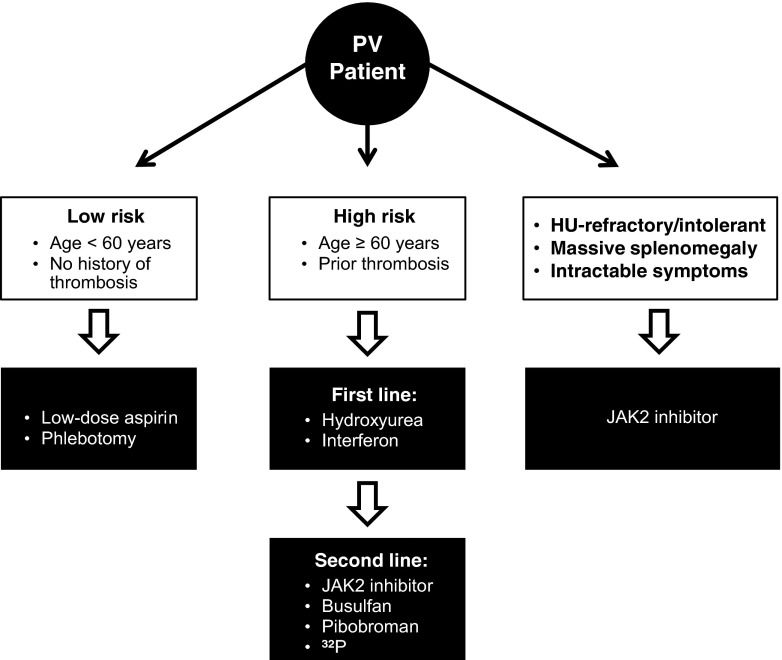
Algorithm for the treatment of polycythemia vera. *HU* hydroxyurea, *PV* polycythemia vera

Initial treatment depends on the risk stratification of the patient, which is shaped by his or her risk of thrombosis [18, 37, 38] and is not designed to estimate survival or the risk of leukemic/fibrotic transformation (Fig. 1) [37]. Patients can be stratified in “high-risk” or “low-risk” categories according to whether they are older or younger than 60 years and have a history of thrombosis. Low-risk patients have zero risk factors; high-risk patients have one or two risk factors [37, 38]. An “intermediate-risk” category that includes younger patients with coexisting cardiovascular risk factors in the absence of previous thrombosis has been proposed but has not been formally evaluated [24, 38]. Leukocytosis and *JAK2* V617F allele burden have been identified as novel thrombotic risk factors but have not been confirmed as such yet [10, 24, 38]. In support of leukocytosis being a risk factor, leukocytosis at PV diagnosis has been associated with patients having a higher risk of developing arterial thrombosis and acute leukemia, with both of these complications resulting in a shorter survival [26, 39]. Additionally, leukocytosis was found to be an independent risk factor in the European Collaboration on Low-Dose Aspirin in Polycythemia (ECLAP) study [40], and furthermore, persistence of leukocytosis despite treatment with HU was associated with a higher risk of hematologic transformation and shorter survival [41]. Unlike leukocytosis, the influence of *JAK2* V617F on thrombotic risk is not clear. Studies have shown that patients harboring a >75 % *JAK2* V617F allele burden are at higher relative risk of developing major cardiovascular and thrombotic events [42]. However, a study by Tefferi and colleagues found no correlation between major cardiovascular events and *JAK2* V617F allele burden [43]. Additionally, age >65 years, male sex, and leukocytosis >10 × 10^9^/L at diagnosis are all associated with a significantly shorter survival [40, 41]. Interestingly, when the different items included in the composite European LeukemiaNet (ELN) response definition were individually considered, being in sustained response as related to PV-related symptoms, spleen size by palpation, Hct, or WBC count was not associated with any significant reduction in the incidence rate of vascular events [41].

Low-risk patients (aged < 60 years and with no prior history of thrombosis) are treated with low-dose aspirin and phlebotomy [3, 37]. The efficacy and safety of low-dose aspirin (100 mg daily) were assessed in the ECLAP double-blind, placebo-controlled, randomized clinical trial (*N* = 518) [44]. A follow-up of 3 years showed a significant reduction in cardiovascular death, nonfatal myocardial infarction, nonfatal stroke, and major venous thromboembolism; major bleeding was not significantly increased. Low-dose aspirin can be used alone (generally in patients with early-stage PV) or can be used in combination with phlebotomy. Phlebotomy lowers Hct values, thus reducing blood hyperviscosity [3], and should be continued until Hct reaches <45 % [31]. Cytoreductive therapy should also be considered for low-risk patients who cannot tolerate phlebotomy, still have severe disease-related symptoms or progressive splenomegaly, or have platelet counts >1500 × 10^9^/L or progressive leukocytosis [13]. The addition of cytoreductive therapy is also recommended for patients at high risk for vascular complications.

For high-risk patients, HU or interferon alpha (IFN-α) are the first-line treatment recommendations [13, 37]; however, IFN-α is not licensed for treatment of PV in most European countries. HU is a cytoreductive agent that decreases the production of all cell lines produced in the bone marrow. HU is useful in controlling PV-related symptoms, splenomegaly, leukocytosis, thrombocytosis, and Hct [41]. However, HU-treated patients can eventually become resistant or experience unacceptable adverse effects (HU intolerance), including skin ulcers, a reduction in blood cells, gastrointestinal problems, oral ulcers, stomatitis, hyperkeratosis, or actinic keratosis. IFN-α has been shown to have antiproliferative effects on hematopoietic precursor cells, induce cytogenetic remissions, and reduce *JAK2* V617F allele burden in patients with MPNs [13]. Unfortunately, intolerable adverse effects, including flu-like symptoms, fatigue, and neuropsychiatric symptoms, and autoimmune problems, such as thyroiditis, have limited its use in PV [13, 24]. Furthermore, prolonged evaluation of IFN-α has been quite difficult because approximately 25 to 40 % of patients with PV treated with IFN in clinical trials discontinued treatment within 1 to 2 years due to adverse effects [45, 46]. Other second-line cytoreductive therapy choices for patients who experience resistance or intolerance to HU include busulfan, pipobroman, or ^32^P [13]. These agents, however, have been linked to potential leukemogenicity [47] and are usually reserved for elderly patients (aged ≥70–80 years) or those with advanced disease, in whom the risk of thrombosis outweighs the risk of AML/myelodysplastic syndromes.

## Unmet medical needs with current strategies

Although some patients respond well to current therapies, effective and well-tolerated treatments are still lacking for both low- and high-risk patients. Low-dose aspirin and phlebotomy help manage the disease, but low-risk patients still have an incidence of vascular events of 2.5 per 100 patients per year (approximately double that of a normal control population) [48]. In some patients, long-term treatment with phlebotomy leads to patient noncompliance and clinical intolerance and may result in iron deficiency, which very frequently leads to fatigue and could potentially lead to reactive thrombocytosis [49, 50]. Additionally, treatment with phlebotomy alone (vs either ^32^P or chlorambucil plus phlebotomy) was associated with a relatively high risk of thrombosis (23 %) in the Polycythemia Vera Study Group 01 study [16, 49], although this risk may be outweighed by aggressively targeting Hct <45 %, as seen in the more recent CYTO-PV study [31]. Among patients treated with HU, approximately one in four will develop resistance (≈11 %) or intolerance (13 %) to this agent, which eventually leads to an increased risk of death [13, 41]. Others who are treated with IFN are unable to continue treatment due to the intolerable adverse effects associated with this therapy [13, 24]; even 20 to 30 % of patients treated with PEG-IFN develop IFN intolerance during long-term therapy [45, 51].

Clinical experience indicates that a large proportion of patients (20–60 %) remain on HU therapy despite lack of response and intolerance. HU resistance leads to an increased risk of death and transformation to MF [41], highlighting the importance of moving these patients to second-line therapies or enrollment in clinical trials. Thus, a definition of HU resistance/intolerance is essential. To this end, specific criteria of HU intolerance/resistance in patients with PV have been proposed by the ELN Working Group (Table 2) [52]. As molecularly targeted therapies become available for the management of patients with MPN, it will become necessary to identify those who (a) will derive the most benefit and (b) are resistant to or intolerant of existing treatment options.

**Table 2:** Definition of resistance to/intolerance of HU in patients with PV

After 3 months of ≥2 g/day of HU, any one of the following:	OR	At the lowest dose of HU required to achieve a complete or partial response, ^a^ any one of the following:	OR	At any dose of HU
• Need for phlebotomy to keep Hct <45 %		• ANC <1.0 × 10^9^/L		• Presence of leg ulcers or other unacceptable HU-related nonhematologic toxicities (e.g., mucocutaneous manifestations, gastrointestinal symptoms, pneumonitis, or fever)
• Uncontrolled myeloproliferation (i.e., PLT count >400 × 10^9^/L and WBC count >10 × 10^9^/L)		• PLT count <100 × 10^9^/L		
• Failure to reduce massive^b^ splenomegaly by >50 % by palpation or resolve splenomegaly-related symptoms		• Hgb <100 g/L		

From [53]

*ANC* absolute neutrophil count, *Hct* hematocrit, *Hgb* hemoglobin, *HU* hydroxyurea, *PLT* platelet, *PV* polycythemia vera, *WBC* white blood cell

^a^Complete response was defined as Hct <45 % without phlebotomy, platelet count ≤400 × 10^9^/L, WBC count ≤10 × 10^9^/L, and no disease-related symptoms. Partial response was defined as Hct <45 % without phlebotomy or response in ≥3 other criteria

^b^Spleen extending >10 cm from the costal margin

## New treatment options

With the development of several new therapies, including targeted agents, standardized criteria for the interpretation and comparison of clinical trials became imperative. In 2013, the ELN and the International Working Group-Myeloproliferative Neoplasms Research and Treatment developed a set of response criteria to be used in clinical trials of new agents (Table 3) [53]. The proposed criteria incorporate clinical, hematologic, and histologic response assessments and evaluate the long-term effects of new and experimental drugs. These criteria are crucial for assessing therapeutic outcomes in patients treated with novel agents and for the approval process of these agents by regulatory agencies.

**Table 3:** Response criteria for PV

	Criteria
Complete remission	
A	Durable^a^ resolution of disease-related signs including palpable hepatosplenomegaly, large symptom improvement,^b^ AND
B	Durable^a^ peripheral blood count remission, defined as Hct <45 % without phlebotomy; platelet count ≤400 × 10^9^/L, WBC count <10 × 10^9^/L, AND
C	Without progressive disease and absence of any hemorrhagic or thrombotic event, AND
D	Bone marrow histologic remission defined as the presence of age-adjusted normocellularity and disappearance of trilinear hyperplasia and absence of reticulin fibrosis > grade 1
Partial remission	Achievement of A, B, and C without bone marrow histologic remission defined as persistence of trilinear hyperplasia
Molecular response^c^	
	Complete response: eradication of a preexisting abnormality
	Partial response^d^: a ≥50 % decrease in allele burden
No response	Any response that does not satisfy partial remission
Progressive disease	Transformation into post-PV MF, MDS, or acute leukemia

Republished with permission of the American Society of Hematology, from Barosi G et al. [53]; permission conveyed through Copyright Clearance Center, Inc.

*Hct* hematocrit, *MDS* myelodysplastic syndrome, *MF* myelofibrosis, *PV* polycythemia vera, *WBC* white blood cell

^a^Lasting at least 12 weeks

^b^Large symptom improvement (≥10-point decrease) in the Myeloproliferative Neoplasm-Symptom Assessment Form Total Symptom Score

^c^Molecular response is not required for assignment as complete response or partial response; it requires analysis in peripheral blood granulocytes

^d^Partial response applies only to patients with at least 20 % mutant allele burden at baseline

### JAK2 inhibitors

The discovery of *JAK2* V617F as the underlying mutation in PV has led to the development of several molecularly targeted therapies focusing on the inhibition of JAK2. The JAK2 inhibitors have demonstrated great activity in patients with MF. Patients with PV who are resistant to or intolerant of HU or IFN and/or are experiencing intractable pruritus, severe constitutional symptoms, or marked splenomegaly might benefit greatly from treatment with a JAK inhibitor, as opposed to conventional therapy [37].

The first reported results of a JAK2 inhibitor for the treatment of PV were those from the phase 2 study of lestaurtinib in patients with PV/ET. Lestaurtinib has been shown to inhibit proliferation and JAK2/STAT5 signaling in cells from patients with myeloproliferative disorders (IC_50_ = 1 nM in vitro) in preclinical studies [54]. The ability of lestaurtinib 80 mg twice daily to decrease *JAK2* V617F allele burden in patients with PV (*n* = 27) or ET (*n* = 12) was examined in a phase 2, open-label, multicenter study (NCT00586651) [55]. The primary endpoint, a ≥15 % reduction in *JAK2* V617F allele burden in 15 % of patients, was not met. Lestaurtinib modestly reduced *JAK2* V617F allele burden and reduced spleen size in a subset of patients. Every patient had ≥1 adverse event (AE), most commonly gastrointestinal (95 %), 15 patients (38 %) experienced serious AEs, and 23 (59 %) withdrew due to AEs. This study highlighted the need for further studies of JAK2 inhibition in the treatment of PV and/or the development of other JAK2 inhibitors.

In 2011, ruxolitinib, a potent JAK1/JAK2 inhibitor, was approved by the US Food and Drug Administration (FDA) for the treatment of MF and later by the European Medicines Agency for the treatment of splenomegaly and MF-related symptoms [56–58]. These approvals were based on the results from two phase 3 Controlled Myelofibrosis Study with JAK Inhibitor Therapy (COMFORT) studies [56–58] showing that ruxolitinib was generally well tolerated and demonstrated rapid and durable clinical benefits, as well as a survival advantage. Given ruxolitinib’s efficacy and safety profile in MF and its activity as a JAK inhibitor, studies of its effects in patients with PV have begun. In preclinical studies, ruxolitinib inhibited erythroid colony formation from cells derived from patients with PV as well as growth-factor-independent colony formation, a unique characteristic of PV and other MPNs [59]. Results from a phase 2, open-label, dose-ranging study (Incyte; *N* = 34) suggested that ruxolitinib was well tolerated and achieved rapid and durable clinical responses in patients with PV who were resistant/intolerant to HU [60]. Response was assessed using modified ELN criteria, which included a reduction in Hct to <45 % without phlebotomy, resolution of palpable splenomegaly, normalization of WBC and platelet counts, and reduction in PV-associated symptoms. Response was achieved in 97 % of patients by week 24 (median duration of exposure, 155 weeks) and was durable, with 85 % maintaining response for 48 weeks. Ruxolitinib improved PV-associated symptoms, including pruritus, night sweats, and bone pain within 4 weeks of treatment initiation. Anemia and thrombocytopenia (primarily grade 1) were the most common AEs.

Based on the promising results from the phase 2 dose-ranging study, the first phase 3 trial of a JAK inhibitor (ruxolitinib) in the treatment of PV, Randomized Study of Efficacy and Safety in Polycythemia Vera with JAK Inhibitor INCB018424 Versus Best Available Care (RESPONSE; ClinicalTrials.gov identifier, NCT01243944), was initiated; results were recently reported [61]. RESPONSE is an open-label, randomized, phase 3 trial comparing the efficacy and safety of ruxolitinib versus best available therapy (BAT) in patients with PV who were resistant to or intolerant of HU by modified ELN criteria. Patients were required to have splenomegaly and need phlebotomy for adequate Hct control. The primary endpoint was a composite of the percentage of patients who achieved both Hct control (defined as the lower of Hct <45 % that was ≤3 points lower than baseline or Hct ≤48 %) from week 8 to week 32 and a ≥35 % reduction in spleen volume from baseline at week 32. Overall, 21 % of patients randomized to ruxolitinib (vs 1 % of BAT-treated patients) achieved the primary endpoint (*P* < 0.0001), with 77 % meeting at least one component. More patients in the ruxolitinib arm had decreases from baseline in spleen volume compared with those in the BAT arm (71.8 vs 33.0 %), with 38 % (vs 1 % in the BAT arm) achieving a ≥35 % reduction in spleen volume. Similarly, higher proportions of patients in the ruxolitinib arm achieved Hct control (60 vs 20 %). In addition, significantly more patients who received ruxolitinib achieved complete hematologic remission (defined as Hct control, platelets ≤400 × 10^9^/L, and WBC count ≤10 × 10^9^/L) at week 32 compared with those who received BAT (23.6 vs 8.9 %; *P* = 0.0034). Although it was not a predefined efficacy assessment in the study, the rate of thromboembolic events was lower in patients receiving ruxolitinib than in patients treated with BAT (1 vs 6). Treatment with ruxolitinib also resulted in great improvements in symptoms, as assessed by the MPN-SAF questionnaire. Overall, ruxolitinib was generally well tolerated and had a safety profile consistent with that seen in the phase 3 COMFORT studies [56, 57], suggesting that ruxolitinib may be a potential new treatment option for patients with PV who are classified as resistant to or intolerant of HU according to modified ELN criteria.

Given that not all patients with PV present with splenomegaly, ruxolitinib is also being evaluated in the RESPONSE 2 trial [62]. RESPONSE 2 is a phase 3b, open-label, randomized (1:1) study comparing the efficacy and safety of ruxolitinib with BAT in patients with HU-resistant/intolerant PV who have a nonpalpable spleen and thus were not eligible for the RESPONSE trial. Another ongoing phase 3 study evaluating ruxolitinib for the treatment of PV is the Randomized Switch Study from Hydroxyurea to Ruxolitinib for RELIEF of Polycythemia Vera Symptoms (RELIEF) trial (Incyte; NCT01632904), a randomized (1:1), multicenter, double-blind, double-dummy, phase 3 switch study evaluating the efficacy and safety of ruxolitinib vs HU for the control of disease-related symptoms in patients with PV currently reporting symptoms on HU monotherapy [63]. The primary endpoint is the proportion of patients with a ≥50 % reduction from baseline in symptoms including tiredness, itching, muscle ache, night sweats, and sweating while awake at week 16.

Momelotinib (CYT387), another JAK inhibitor currently under evaluation, is a JAK1/JAK2 inhibitor that has demonstrated clinical improvement in MF in a phase 1/2 clinical study [64]. Treatment with momelotinib resulted in a durable reduction of splenomegaly and the achievement of sustained red blood cell transfusion independence in a substantial number of participants in this study. Based on these results, a phase 2, open-label, randomized study evaluating the safety and efficacy of momelotinib in patients with PV or ET is currently underway (NCT01998828). Patients must be intolerant of, resistant to, or refuse current available treatment for PV. The primary endpoint is an overall response rate defined as the proportion of participants who experience all of the following for ≥4 weeks during the treatment period: Hct <45 % in the absence of phlebotomy, WBC count <10 × 10^9^/L, platelet count ≤400 × 10^9^/L, and resolution of palpable splenomegaly.

One other JAK inhibitor still in early development is the selective JAK2 inhibitor LY2784544, which has demonstrated dose-dependent selectivity for the mutated *JAK2* V617F over wild-type *JAK2* [65]. LY2784544 was tested in a phase 1 study (NCT01134120) in 38 patients with *JAK2* V617F-positive MF (*n* = 31), ET (*n* = 1), or PV (*n* = 6). The primary objectives were to determine the safety and tolerability of LY2784544 and to define a recommended dose for further study. Of the six patients with PV, three achieved a clinicohematologic partial response at a dose of 120 mg per day. The most frequently reported drug-related AEs across all grades were diarrhea (44 %); nausea (29 %); increased creatinine (21 %); and anemia, vomiting, and fatigue (9 % each); there were no grade 4 AEs. The authors concluded that the results support ongoing phase 2 testing at a daily dose of 120 mg.

### Histone deacetylase inhibitors

Another class of targeted therapy being explored is histone deacetylase (HDAC) inhibitors, which inhibit proliferation of tumor cells by inducing cell cycle arrest, differentiation, and/or apoptosis [66]. Givinostat has specificity for *JAK2* V617F-mutated cells and has been tested in a pilot phase 2 study in patients with HU-resistant/intolerant *JAK2* V617F-positive PV (*n* = 12) and ET (*n* = 1) [67]. Givinostat was well tolerated, with no grade 4 toxicities reported. Overall, 75 % of patients had a reduction in splenomegaly and 54 % had a clinical response after 12 weeks on treatment. Givinostat was later evaluated in a multicenter, open-label phase 2 study in patients with PV (*n* = 44) who showed no response when treated with the maximum tolerated doses of HU. Patients were treated with givinostat (50 or 100 mg/day) in combination with HU at the maximum tolerated dose. The combination of givinostat and HU was well tolerated, and after 12 weeks of treatment, complete or partial response was observed in 55 and 50 % of patients receiving 50 or 100 mg givinostat, respectively. Improvements in pruritus were also observed (64 and 67 %) [68].

Other HDAC inhibitors have not been as well tolerated. Vorinostat was tested in a nonrandomized, open-label phase 2 trial enrolling patients with PV (*n* = 44) and ET (*n* = 19) [69]. Overall, 72 % of patients had a response, but 44 % of patients discontinued treatment due to AEs.

### Pegylated interferon

Newer pegylated formulations of IFN (PEG-IFN), which are better tolerated and allow for less frequent administration, have renewed interest in IFN as a therapeutic option for patients with PV, including those who are refractory or resistant to HU. In addition to having a more favorable toxicity profile than HU and IFN, PEG-IFN treatment has been associated with high rates of hematologic and molecular responses that may prevent evolution to MF and AML. A 2008 phase 2 study (*N* = 37) found that all patients receiving PEG-IFN had a hematologic response and a reduction in *JAK2* V617F allele burden [45]. Similarly, another study (*N* = 40) demonstrated an overall hematologic response rate of 80 %, with a 14 % complete molecular response [70]. However, PEG-IFN is contraindicated in patients with thyroid and psychiatric disorders, and data on its prevention of thromboembolic events are limited. PEG-IFN is being tested in two phase 3 trials for the treatment of PV. The first trial, sponsored by the Myeloproliferative Disorder Research Consortium, is a randomized open-label study evaluating the safety, tolerability, and efficacy of PEG-IFN-2a (Pegasys) vs HU in patients with high-risk PV or ET (NCT01259856) [71]. The primary outcome will be a comparison of hematologic rates between the two study arms. Pegylated Interferon Alpha-2b Versus Hydroxyurea in Polycythemia Vera (PROUD-PV; NCT01949805), a second phase 3 study, is currently evaluating AOP2014, a novel PEG-IFN, in patients with PV. This trial will compare the safety and efficacy of AOP2014 with HU in patients who either have not had prior exposure to HU or have had no response to prior HU treatment [72]. The primary endpoint is the disease response rate (Hct < 45 % without phlebotomy, platelets <400 g/L, leukocytes <10 g/L, and normal spleen size) at 12 months. This study is currently recruiting patients, with an estimated enrollment of 256 patients.

## Conclusions

PV is the most common of the MPNs. Patients experience debilitating pruritus and fatigue and develop new symptoms, such as splenomegaly, as the disease progresses, while facing the risk of major thrombotic events. Although PV is associated with increased mortality, many patients have a long median survival, highlighting the importance of effective and well-tolerated therapy. Patients have limited therapeutic options, and many must pursue inadequate treatment accompanied by intolerable adverse effects and the risk of progression to MF or hematologic transformation. The discovery of *JAK2* mutations as the underlying molecular basis for PV has greatly increased our understanding of the pathogenesis of PV and has allowed for the development of targeted therapies. Already, studies are assessing the clinical benefits of JAK2 inhibitors and are showing promising results for the treatment of this debilitating disease. Further studies will most likely focus on which patients with PV will benefit most from the use of targeted therapies and how these new therapies compare with the current therapy standards. The best therapy for each patient will be one that is well tolerated while improving symptoms and quality of life, and in this regard, targeted therapies will be valuable tools.
